# *CHLORELLA* should not be a long noncoding RNA: it’s already an alga!

**DOI:** 10.1093/ismeco/ycag117

**Published:** 2026-04-25

**Authors:** David Roy Smith, Breno Dupin, Matheus Sanita Lima

**Affiliations:** Department of Biology, Western University, London, ON N6A 5B7, Canada; Department of Biology, Western University, London, ON N6A 5B7, Canada; Department of Biology, Western University, London, ON N6A 5B7, Canada

**Keywords:** *Chlorella*, lncRNA, *Arabidopsis*, gene name, species name, chloroplast

*The only thing worse than bad health is a bad name.* [1] *— Gabriel Garcia Marquez*

We read with enthusiasm the recent article by Kang *et al*. [2] describing an *Arabidopsis* nuclear-encoded, chloroplast-targeted long noncoding RNA (lncRNA) involved in chloroplast regulation. This lncRNA appears to act as a developmental switch within chloroplasts, mediating the transition from early biogenesis to late degeneration during leaf ageing [2]. These data are exciting because they show that anterograde signalling between the nucleus and chloroplast is more complicated and nuanced than previously thought [3].

However, one aspect of this novel *Arabidopsis* noncoding RNA struck us as odd: its name. The authors are calling it *CHLORELLA*, short for *CHLOROPLAST-RELATED LONG NONCODING RNA*. At first glance, this name sounds fine. Except for one important detail—it is already taken! As many microbial ecologists are aware, there is a genus of green alga called *Chlorella*. To be fair, *Chlorella* is not as famous as the research superstar *Arabidopsis*. But *Chlorella* species have been model research organisms for over a century [4], and once were the ‘workhorses’ of chloroplast studies along with other micro algae and crop species [5]. Melvin Calvin’s work on chloroplast carbon dioxide assimilation [6], for example, resulted in a Nobel prize in 1961. Nobel laureate Otto Heinrich Warburg (1931) also used *Chlorella* as his primary model for investigating the mechanisms of photosynthesis [7]. More recently, *Chlorella* species have taken centre stage in microbial ecology studies (published in ISME) investigating long-term symbioses [8] and interspecies competition [9].

We believe naming an important land plant ncRNA after a model green alga is not only confusing but counterproductive to research on microbial eukaryotes. *CHLORELLA* has not yet been identified in *Chlorella*, or any other green alga. But *Chlorella* has a chloroplast, so there is a chance it might have *CHLORELLA*; it would be even more perplexing if *Chlorella* lacked *CHLORELLA*. You get the point—gene nomenclature is a serious issue in the omics era [10].

To the best of our knowledge, *CHLORELLA* is the first published case of a name collision between an ncRNA and a valid genus name. As other cautionary tales from the media [11, 12] and primary literature can attest [13], the lack of standardization in gene nomenclature and naming practices are an obstacle to comparative omics studies. Indeed, a poorly chosen name can confound the evolutionary history of a gene (or any molecular locus) [13], as is epitomized by the name *CHLORELLA*. Moreover, unconventional gene names hinder text-mining efforts for ecological and biomedical research [14]. It is interesting to ponder how large language models will treat *CHLORELLA* the ncRNA when its semantic network is tied up with *Chlorella* the alga (and vice versa). The English linguist J. R. Firth said it best: ‘You shall know a word by the company it keeps’ [15].

Gene naming practices are of particular importance to biologists, including microbial ecologists, tackling research questions through a comparative approach. Just recently, for our own comparative genomics endeavours, we had to develop a bionformatics `gene dictionary tool' (GDT for short) to address inconsistencies in gene names [16]. This is precisely why international consortia and committees, such as the Human Gene Nomenclature Committee (HGNC), have issued clear gene nomenclature guidelines. The HGNC stresses that ‘gene symbols should not spell out commonly used words’ [17], and it considers ‘[gene] symbols that affect data handling and retrieval’ one of the few cases for which gene symbols might be suitable for a change [18]. For instance, the gene names SEPT1 and MARCH1 had to be altered because they could be mistaken for dates in conventional spreadsheets. The genes WARS and CARS also had to be renamed because of their similarity to common English words. And the list goes on. Perhaps the most notorious examples of ill-advised gene names are Pokémon, Velcro, and lunatic fringe [11, 12], all of which had to be changed, in some cases under the threat of litigation. Name collision is also a great concern for the naming of species. In fact, how to handle and avoid nomenclatural confusion (greatly derived from homonyms) is one of the core goals of the International Code of Nomenclature for algae, fungi, and plants [19].

Regarding lncRNAs specifically, Mattick *et al*. [20] wrote that ‘unless a lncRNA is antisense to a protein-coding gene, we recommend naming lncRNAs for their own sake with allusion to a discerned characteristic or function (as has been traditional for proteins) (…) If no biological context is available, we recommend naming the lncRNA according to the GENCODE system’. Pointedly, TAIR (The Arabidopsis Information Resource) states that: ‘Gene names should convey some meaning as to the function of the gene product’ [21]. As it stands, the naming of *CHLORELLA* does not follow these recommendations.

*Chlorella* is a kind-hearted phototroph and not litigious by nature, so we don’t think Kang *et al*. [2] have much to worry about. But this freshwater alga has had a hard time of late apropos its name. It’s a touch overused. Walk into any health food store and you’ll be bombarded with wellness products boasting the word Chlorella in their titles, from vitamins to creams to shampoos. There are also lines of chlorella clothing and sneakers. And some ‘health’ gurus are even promoting Chlorella injections. Given all this, Kang *et al*. [2] should consider renaming this fascinating functional noncoding RNA to something more redolent of *Arabidopsis* or land plants in general, following the guidelines quoted above. In no way, however, do we want this small criticism to detract from the work of Kang *et al*. [2]. In a recent article, we speculated how lncRNAs could participate in anterograde/retrograde signalling and even organelle nucleoid topology [22]. In other words, it would be an understatement to say we have been eagerly awaiting the identification of a bona fide functional noncoding RNA in a chloroplast system. Thankfully one has arrived. But to be clear: it is not an alga.

## Data Availability

Not applicable.
